# Highly Sensitive Electrochemical Detection of Pomalidomide Using a Novel NiCuSe_2_/MnCoFe LTH Nanocomposite Sensor: Application to Real Sample Analysis

**DOI:** 10.1021/acsomega.5c00694

**Published:** 2025-06-20

**Authors:** Wiem Bouali, Asena Ayse Genc, Nevin Erk, Hassan Elzain Hassan Ahmed, Mustafa Soylak

**Affiliations:** † Department of Analytical Chemistry, 37504 Ankara University, Faculty of Pharmacy, 06560 Ankara, Turkey; ‡ Health Sciences, Ankara University, The Graduate School of the, 06110 Ankara, Turkey; § Department of Chemistry, 52958 Erciyes University, Faculty of Sciences, 38039 Kayseri, Turkey; ∥ Technology Research & Application Center (TAUM), Erciyes University, 38039 Kayseri, Turkey; ⊥ Turkish Academy of Sciences (TUBA), Cankaya, Ankara 06700, Turkey

## Abstract

A novel electrochemical sensor for the quantification of Pomalidomide (POMA) is introduced in the present work, based on a glassy carbon electrode (GCE) modified with a unique NiCuSe_2_/MnCoFe layered triple hydroxide (LTH) composite. In this regard, the novel NiCuSe_2_/MnCoFe LTH composite was synthesized and characterized using various structural and morphological techniques, confirming its successful formation. The electrochemical properties of the composite were evaluated by cyclic voltammetry (CV) and electrochemical impedance spectroscopy (EIS), providing its high conductivity, superior electrocatalytic activity, and rapid electron transfer capabilities. The NiCuSe_2_/MnCoFe LTH-modified GCE was utilized for the detection and quantification of POMA using differential pulse voltammetry (DPV). The developed sensor exhibited an impressive linear detection range of 0.02–10.3 μM and an ultralow detection limit of 4.7 nM. Furthermore, it demonstrated exceptional selectivity, reproducibility, and repeatability. Finally, the sensor was applied to analyze POMA in some real samples: pharmaceutical capsules, human urine, and human blood serum.

## Graphical abstract

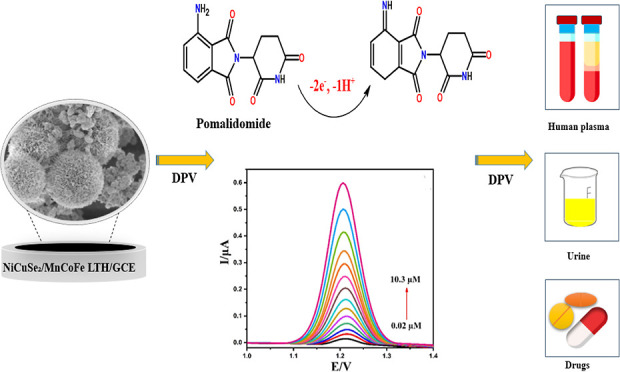

## 1 Introduction

Multiple myeloma (MM) is the second most prevalent malignancy of the hematologic system worldwide, following non-Hodgkin’s lymphoma. With the global rise in the elderly population, the incidence of MM has been steadily increasing, highlighting the critical importance of advancing its treatment.^1^ Pomalidomide, chemically known as (RS)-4-amino-2-(2,6-dioxo-3-piperidinyl)-1H-isoindole-1,3­(2*H*)-dione, is a small-molecule analog of thalidomide developed by Celgene Corporation. Pomalidomide is given orally in the treatment of hematological malignancies and connective tissue disorders.^2^ POMA is reported to be 10 times more potent than lenalidomide and 500 times more potent than thalidomide in inhibiting tumor necrosis factor-alpha (TNF-α).^3^ In February 2013, the U.S. Food and Drug Administration approved Pomalidomide for use in treating relapsed and refractory multiple myeloma.^4^ This approval applies to patients who have undergone at least two prior treatments, such as lenalidomide and bortezomib, and whose disease has progressed either during or within 60 days of completing their most recent therapy.^5^ Pomalidomide has also been investigated for the treatment of different solid tumors, including prostate cancer, small cell lung cancer, pancreatic cancer, and Waldenström’s macroglobulinemia.^2^ Accurate qualitative and quantitative measurement of POMA is essential for ensuring the quality and efficacy of its commercial products. These measurements are crucial for monitoring drug concentrations, ensuring proper dosing, and assessing treatment efficacy in clinical settings.^6^

Various analytical techniques have been utilized to quantify POMA, such as Liquid chromatography–mass spectrometry (LC–MS/MS),^7^ Ultraperformance liquid chromatography–mass spectrometry (UPLC-MS/MS),^8^ spectrofluorometric,^9^ and high-performance liquid chromatography (RP-HPLC).^10^ Although these methods provide accurate results, they come with several limitations such as long analysis times, complex sample preparation, high costs, and the need for skilled operators. These challenges can be addressed by utilizing electrochemical methods, including conductometry, potentiometry, amperometry, and voltammetry. Electrochemical methods offer several advantages, such as low detection limits, reduced costs, quicker sample preparation, and greater flexibility and practicality, making them a more efficient alternative for drug analysis.^11^

Several electroanalytical techniques are available for the electrochemical detection of drugs. Certain methods are particularly suited for detecting drug accumulation, considering the specific electrochemical interactions between the nanomaterials used, the target analytes, and their intended applications. CV and DPV methods are commonly employed in drug detection because of their ability to detect and quantify drugs at low concentrations, especially when coupled with nanomaterial-based sensors.^12^

Recent advancements in electrochemical sensor technology have focused on integrating nanomaterials and polymers to improve sensitivity, selectivity, and detection limits.^13^

LTHs, consisting of three different metal cations within their layers, have recently gained considerable attention as electrocatalysts. Their appeal stems from their unique layered structures, adjustable electronic properties, and exceptional physical and chemical attributes.^14^ LTHs, which are clays with a two-dimensional (2D) structure, have become a subject of considerable interest in the scientific community because of their high surface area, abundant hydroxyl groups, significant positive charges, good catalytic activity, chemical stability, nontoxic nature, nanometer-scale morphology, low cost, and high adsorption capacity.^15^ As a result, layered triple hydroxides (LTHs) typically demonstrate superior electrocatalytic activity when compared to layered double hydroxides (LDHs) . Owing to these advantages, LTHs are widely applied across various fields, including sensors, biomedicine, UV resistance, electrocatalysis, environmental protection, and energy storage.^16^ Recent studies, such as the work by Patil et al., demonstrated the enhanced electrocatalytic activity of NiCoFe LTH by modulating the electronic structure and active sites for efficient applications like urea electrolysis.^17^ Based on these findings, we synthesized MnCoFe LTH, aiming to exploit its similar advantageous properties, including high surface area and tenable electronic features, to improve electrochemical performance in drug detection.

Transition metal selenide (TMSe) materials have recently been identified as a promising category of electrode materials, owing to their significantly enhanced capacitive properties. These improvements can be attributed to their increased number of redox-active sites, superior faradaic reaction performance, excellent electrical conductivity, natural abundance, and notable electrocatalytic activity.^18^ Among these, NiCuSe_2_ has garnered attention for its robust structural integrity, high electronic conductivity, and synergistic effects arising from the incorporation of Ni and Cu in the selenide framework. These characteristics not only improve charge transfer efficiency but also facilitate stable and efficient faradaic processes, making NiCuSe_2_ highly suitable for use in composite systems.^19^ By integrating NiCuSe_2_ with MnCoFe LTH, a hybrid material was designed to leverage the complementary properties of both components, aiming to achieve enhanced electrocatalytic performance, improved sensitivity, and superior selectivity in drug detection applications.

In this study, we utilized a novel NiCuSe_2_/MnCoFe LTH-modified GCE for the sensitive and selective voltammetric determination of POMA. The fabricated sensor was validated for the accurate quantification of POMA in pharmaceutical and biological samples.

## 2 Experimental Section

### 2.1 Apparatus and Chemicals

Information on the apparatus and chemicals used is provided in the Supporting Information.

### 2.2 Synthesis of NiCuSe_2_/MnCoFe LTH Nanocomposite

#### 2.2.1 Synthesis of NiCuSe_2_

The synthesis of NiCuSe_2_ was carried out utilizing a previously developed approach with minor modifications.^20^ 0.58 g of Ni­(NO_3_)_2_·6H_2_O and 0.48 g of Cu­(NO_3_)_2_·3H_2_O were dissolved in 30 mL of deionized water in a beaker and sonicated for 15 min to produce solution A. In a separate beaker, 0.35 g of selenium metal was mixed with 10 mL of hydrazine monohydrate and put in an ultrasonic bath for 15 min to produce solution B. Each solution was stirred separately for 20 min, following which solution A was added to solution B and stirred for an additional 15 min. The resulting mixture was transferred into a 100 mL Teflon-lined hydrothermal autoclave and heated at 170 °C for 48 h in an oven. After cooling, the black product was thoroughly washed several times with deionized water and ethanol, followed by drying at 80 °C for 12 h in an oven (Figure 1i).

**Figure 1:**
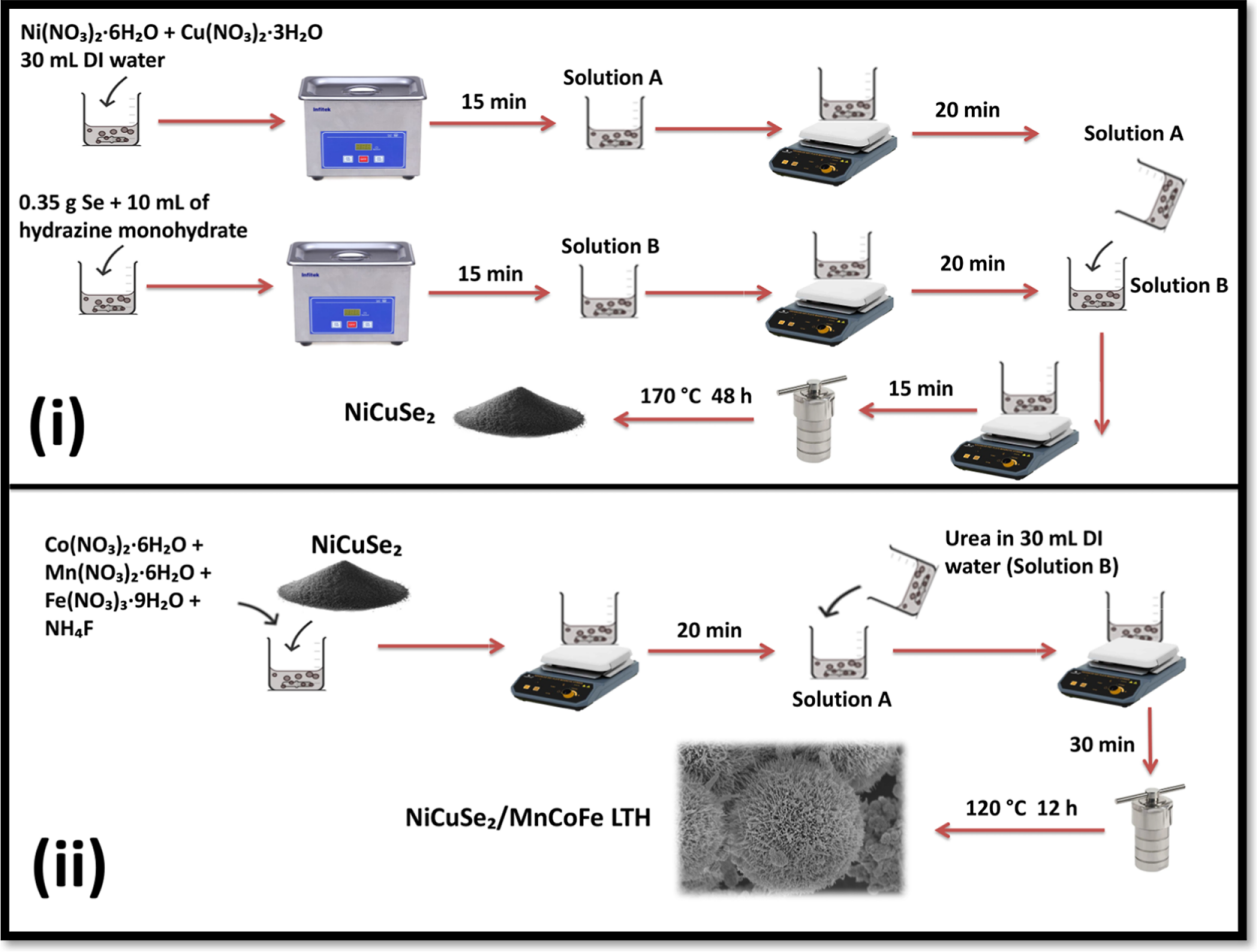
Schematic presentation of the synthesis of (i) NiCuSe_2_ and (ii) NiCuSe_2_/MnCoFe LTH nanocomposite.

#### 2.2.2 NiCuSe_2_/MnCoFe LTH Nanocomposite

To prepare the NiCuSe_2_/MnCoFe LTH nanocomposite, 1.76 g of Co­(NO_3_)_2_·6H_2_O, 0.88 g of Mn­(NO_3_)_2_·6H_2_O, and 0.80 g of Fe­(NO_3_)_3_·9H_2_O were dissolved in 30 mL of deionized water, maintaining a 3:3:1 molar ratio, to form solution A. 1.0 g of synthesized NiCuSe_2_ and 0.40 g of NH_4_F were incorporated into solution A, and the resultant mixture was stirred for 20 min. In a separate beaker, 1.25 g of urea was solubilized in 15 mL of deionized water to produce solution B. The prepared solution (B) was slowly added to solution A, and the final mixture was stirred again for 30 min. The mixture was placed in an 80 mL Teflon-lined autoclave and heated in an oven at 120 °C (12 h) (Figure 1ii). After the reaction mixture was allowed to cool, the resulting NiCuSe_2_/MnCoFe LTH nanocomposite was washed multiple times with ethanol and deionized water to eliminate any impurities. The cleaned product was subsequently dried at 80 °C (12 h) to obtain the final NiCuSe_2_/MnCoFe LTH nanocomposite.

### 2.3 Fabrication of NiCuSe_2_/MnCoFe LTH/GCE and Electrochemical Measurement

The GCE (3 mm diameter) surface was initially polished using an alumina slurry, followed by thorough rinsing with a 1:1 (v/v) mixture of deionized water and ethanol to remove any residual alumina particles. A suspension of NiCuSe_2_/MnCoFe LTH (0.5 mg/mL) was prepared in a water–ethanol mixture, and 3 μL of this suspension was carefully drop-cast onto the cleaned GCE surface. The modified electrode was then subjected to infrared (IR) radiation for 15 min to facilitate drying. This procedure resulted in the successful fabrication of the NiCuSe_2_/MnCoFe LTH/GCE, which was subsequently used for the electrochemical detection of POMA.

Throughout all electrochemical analyses, the working electrode was composed of NiCuSe_2_/MnCoFe LTH/GCE, a platinum wire (Pt) was used as the counter electrode, and Ag/AgCl served as the reference electrode. EIS and CV were performed in a solution containing 5.0 mM [Fe­(CN)_6_]^−3^/^–4^ in 0.1 M KCl. For CV measurements, the potential was scanned from −0.6 to 1.1 V at a rate of 50 mV/s. EIS analyses were conducted over a frequency range of 10–0.1 Hz with an applied potential of 0.1 V. Moreover, the DPV method was utilized with a modulation amplitude of 0.05 V, a modulation time of 0.01 s, and a step potential of 0.005 V.

### 2.4 Preparation of Real Samples for Analysis

The NiCuSe_2_/MnCoFe LTH-modified GCE developed in this study was successfully utilized for the detection of POMA in pharmaceutical capsules, human plasma, and human urine samples. Spiked sample preparation was carried out according to the protocol outlined in our previous study.^21^

## 3 Results and Discussion

### 3.1 Characterization of NiCuSe_2_/MnCoFe LTH Nanocomposite

The FE-SEM images in Figure 2 show the morphological structure of (a–c) NiCuSe_2_ and (d–f) NiCuSe_2_/MnCoFe LTH nanocomposite. Clusters of grains with moderate porosity and low surface roughness are formed by aggregated spherical particles with a smooth, dense structure in pure NiCuSe_2_. This suggests either a strongly defined crystalline or semicrystalline phase. However, the NiCuSe_2_/MnCoFe LTH presents a notable structural change, with nanosheets arranged over the NiCuSe_2_ core and a thin, flower-like structure. These shapes have significant surface roughness, increased porosity, and a hierarchical, spherical network, typical of LTHs. This combination enhances the material’s surface area and structural intricacy, providing more active spots for prospective applications.^14,22^

**Figure 2:**
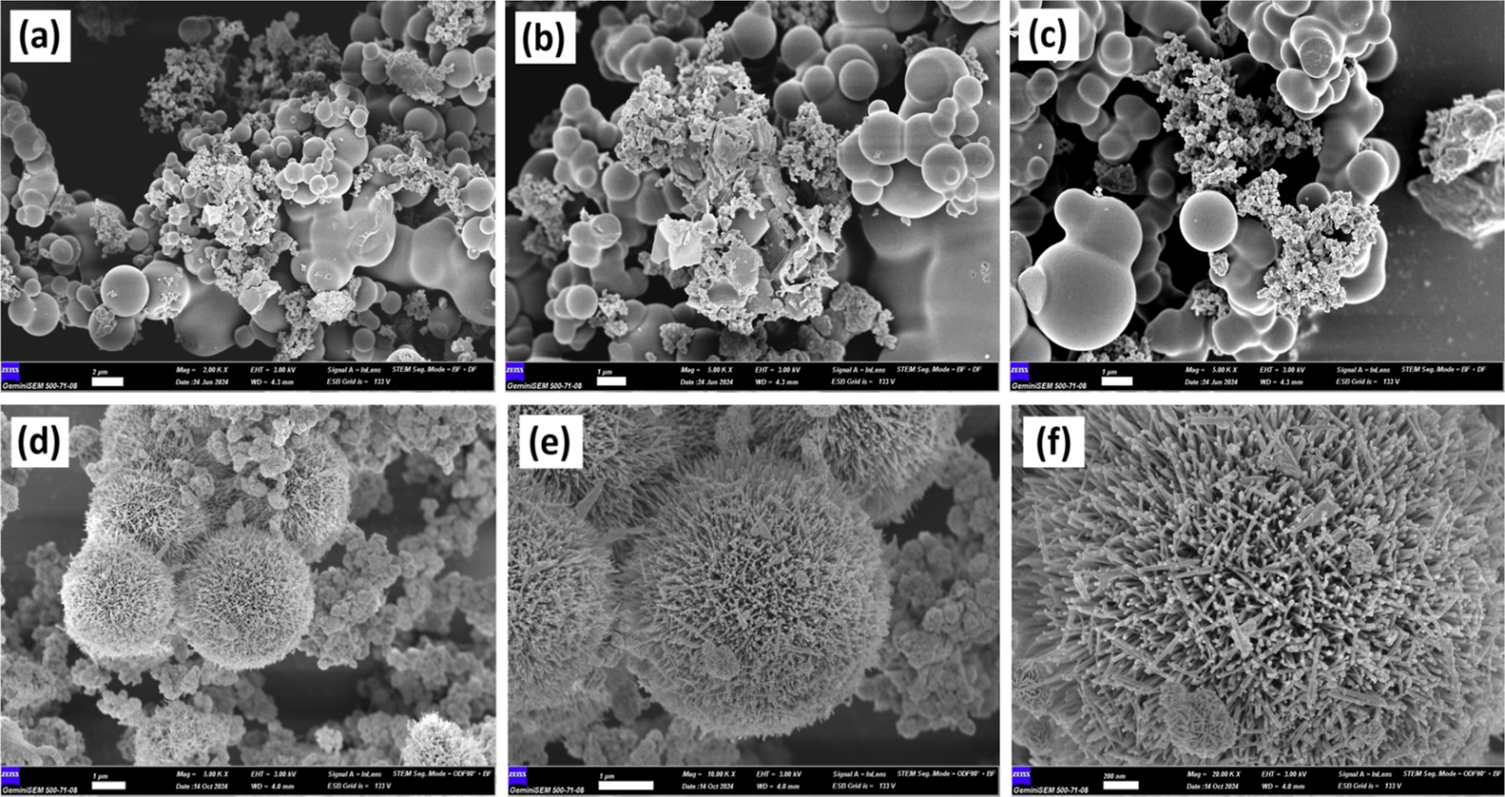
FE-SEM images of NiCuSe_2_ (a–c) and NiCuSe_2_/MnCoFe LTH (d–f).

The SEM–EDX images and elemental composition of (a,b) NiCuSe_2_ and (c,d) NiCuSe_2_/MnCoFe LTH nanocomposite are shown in Figure 3. NiCuSe_2_ exhibited spherical particles with a uniform size distribution and minimal porosity. The elemental composition of NiCuSe_2_ primarily consists of C, O, Ni, Cu, and Se, which confirms the NiCuSe_2_ phase. The high carbon concentration is likely caused by impurities from substrates. The NiCuSe_2_/MnCoFe LTH nanocomposite showed a clear change in structure, which could be seen on the surface by the flower-like structures. The EDX study of the NiCuSe_2_/MnCoFe LTH nanocomposite confirms the presence of Mn, Co, and Fe, along with elevated oxygen levels, matching with the hydroxide characteristics of LTHs.^14^

**Figure 3:**
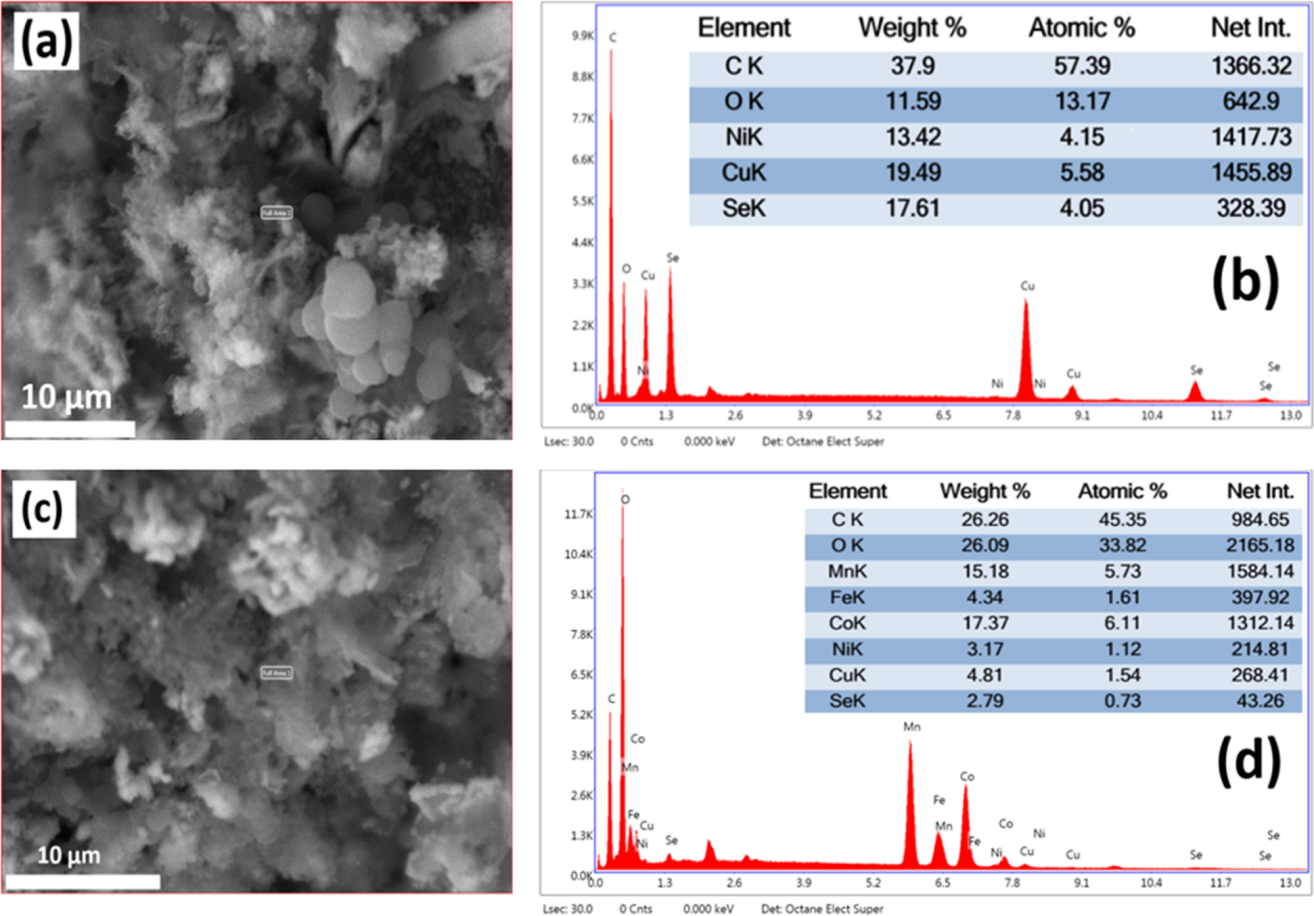
SEM–EDX and the elemental composition of NiCuSe_2_ (a,b) and NiCuSe_2_/MnCoFe LTH (c,d), respectively.

Figure 4 presents the SEM-mapping images of the NiCuSe_2_/MnCoFe LTH nanocomposite. The mapping analysis showed a spatial distribution of key elements (C, Mn, Fe, Co, Ni, Cu, and Se). It shows a uniform and overlapping integration of all elements. It indicates a successful synthesis of the NiCuSe_2_/MnCoFe LTH nanocomposite.

**Figure 4:**
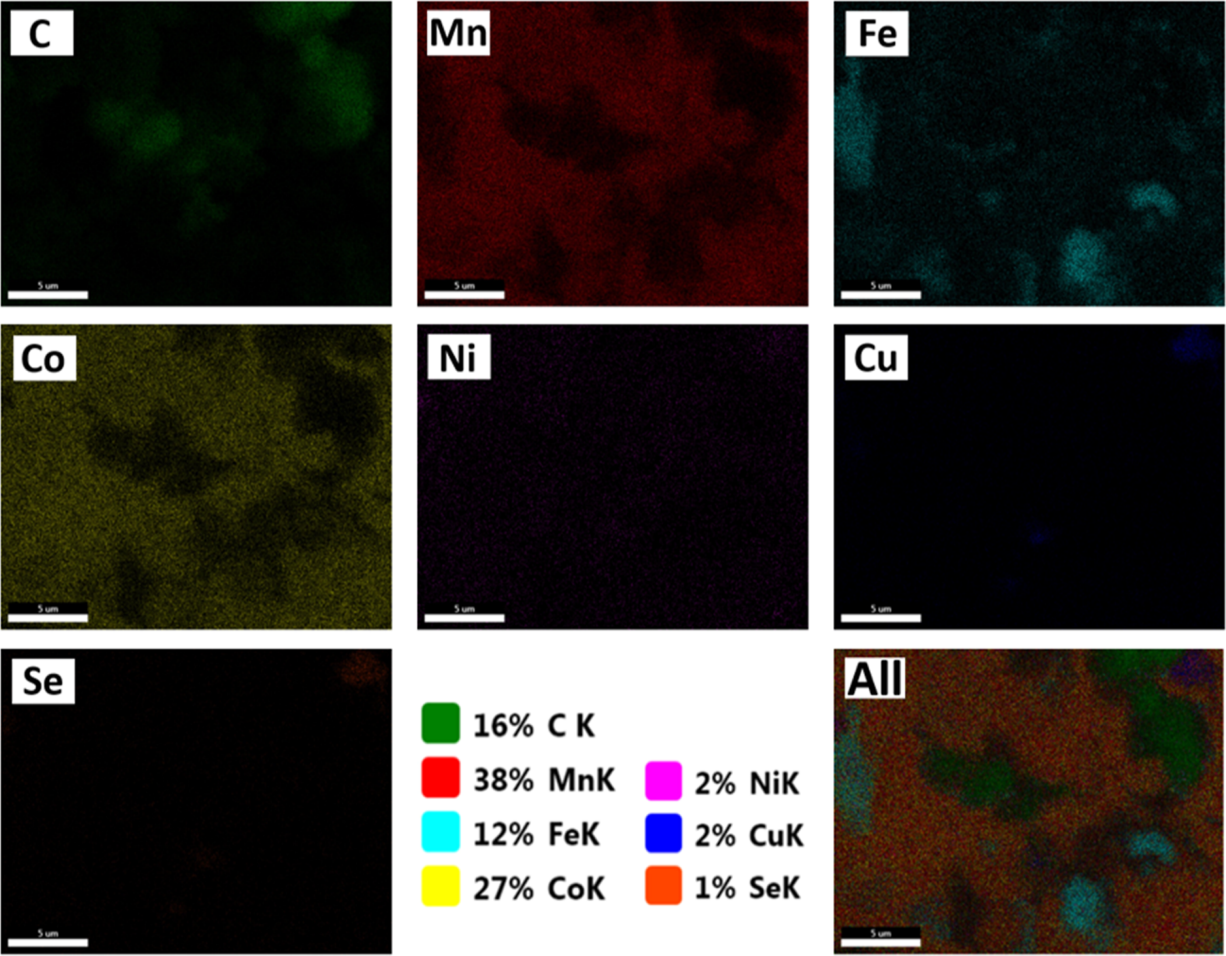
SEM-Mapping images of NiCuSe_2_/MnCoFe LTH.

Significant structural and chemical changes in the NiCuSe_2_/MnCoFe LTH nanocomposite are shown by the FTIR spectra of NiCuSe_2_ and the NiCuSe_2_/MnCoFe LTH nanocomposite (Figure 5). The existence of –OH groups (wide band at 3408 cm^–1^, O–H stretching) and carbonate (CO_3_^=^) as a structural component (peaks at 1353 cm^–1^, asymmetric stretching) is confirmed by the FTIR spectra of MnCoFe LTH. At 770 cm^–1^, metal–oxygen vibrations point to LTH frameworks. Peak shifts indicate mixed-metal interactions. The NiCuSe_2_/MnCoFe LTH spectrum has a significant peak at around 3600 cm^–1^. This peak signifies the existence of –OH associated with the LTH structure, which is not seen in the NiCuSe_2_ spectrum. Both spectra show a small peak around 2900 cm^–1^, suggesting the presence of trace organic contaminants or CH bonds. Strong peaks for C=O and C=C stretching vibrations at 1720 and 1550 cm^–1^, respectively, indicate probably carbon-based residues: the NiCuSe_2_/MnCoFe LTH displays reduced intensity in this area. This indicates changes in the chemical structure after LTH formation. Moreover, the apparent –C–O–C or CH vibration in the NiCuSe_2_/MnCoFe LTH spectrum at 1380 cm^–1^ indicates the existence of carboxylate groups produced by structural changes. The NiCuSe_2_/MnCoFe LTH spectrum shows more clearly the C–O stretching peaks at about 1050 cm^–1^, implying further bonds from the LTH structure. The NiCuSe_2_/MnCoFe LTH spectra show strong metal–oxygen vibrations in the 750–550 cm^–1^ range. These vibrations point to metal–oxygen bonds from Mn, Co, and Fe in the LTH. In this area the NiCuSe_2_ spectrum exhibits reduced intensity and fewer characteristics. The additional peaks in the NiCuSe_2_/MnCoFe LTH spectrum confirm the effective doping of the LTH layer onto the NiCuSe surface.^23^

**Figure 5:**
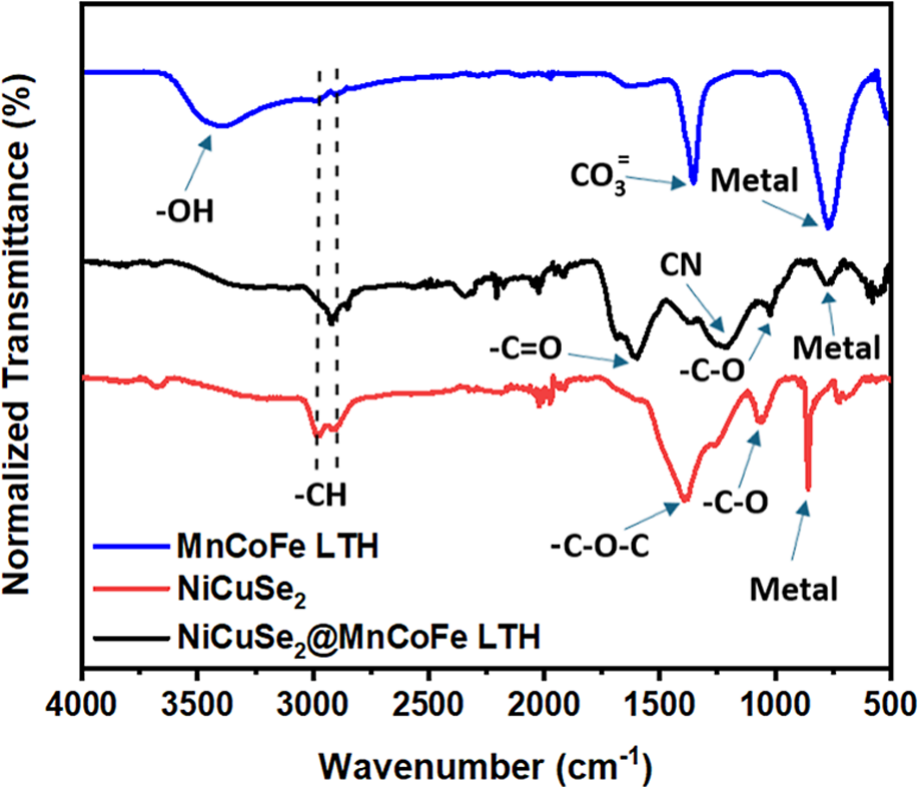
FTIR spectra of NiCuSe_2_ and NiCuSe_2_/MnCoFe LTH nanocomposite.

The XRD study shows the patterns of NiCuSe_2_ and NiCuSe_2_/MnCoFe LTH nanocomposite (Figure 6), each exhibiting unique structural properties. The NiCuSe_2_ displays clear peaks that show a highly crystalline structure, with peaks corresponding to the CuSe (JCPDS 34-171) and NiSe (JCPDS 65-3425 and JCPDS 18-0887) phases.^24^ This demonstrates the successful synthesis of the NiCuSe nanomaterial. The NiCuSe_2_/MnCoFe LTH nanocomposite presents wider and less intense LTH peaks, along with extra peaks from CoFe LDH and MnFe LDH (JCPDS 014-0191) phases.^25^ These demonstrate the effective formation of the LTH layer on the NiCuSe_2_ surface. This coating impairs the long-range crystallinity of the original NiCuSe_2_ material, as seen by the reduced intensity and some larger peaks.^14^ The XRD results validate the crystalline structure of NiCuSe_2_, comprising its CuSe and NiSe components, and underline the successful synthesis of the NiCuSe_2_/MnCoFe LTH nanocomposite as a new nanomaterial.

**Figure 6:**
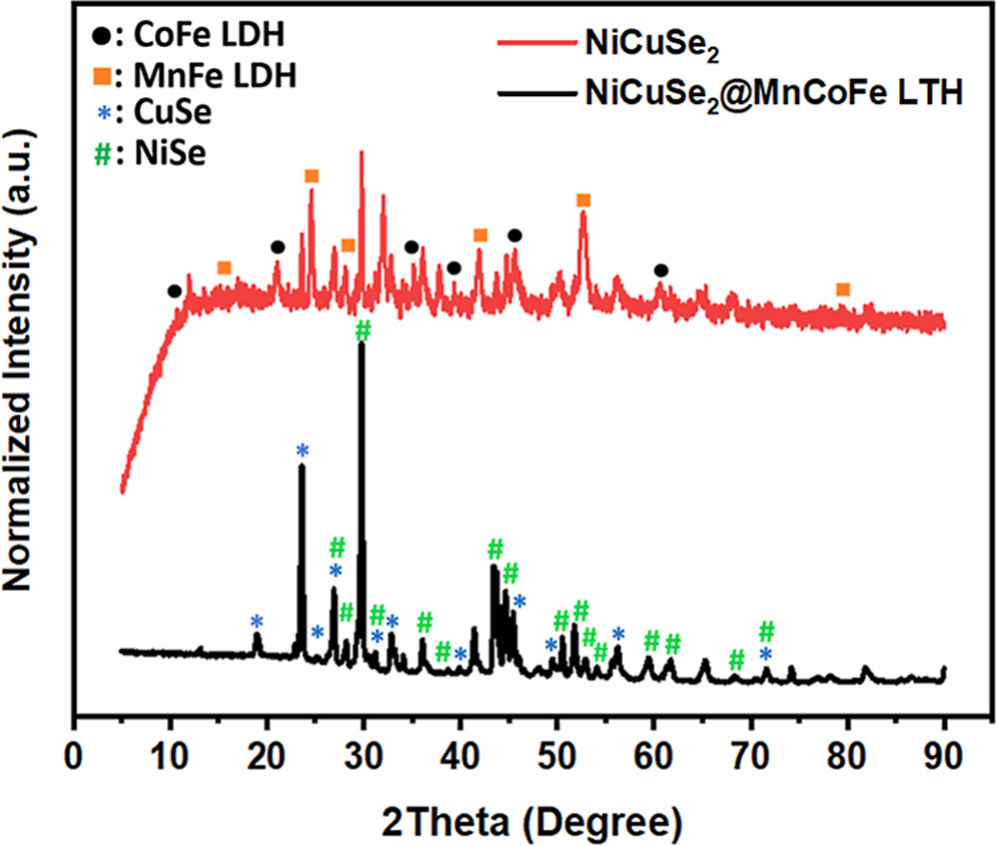
XRD patterns of NiCuSe_2_ and NiCuSe_2_/MnCoFe LTH nanocomposite.

Figure 7 shows the BET analysis of (i) MnCoFe LTH, (ii) NiCuSe_2_, and^26^ NiCuSe_2_/MnCoFe LTH. It shows data about their surface area and pore diameter. MnCoFe LTH has a BET specific surface area of 7.62 m^2^/g, with average pore diameters of 57.5 Å (adsorption) and 73.6 Å (desorption), which indicates a well-defined mesoporous design. The Type IV isotherm and distinct hysteresis loop indicate effective diffusion and stability. On the other hand, NiCuSe_2_ has a slightly reduced surface area of 7.24 m^2^/g and a smaller average pore diameter (26.86 Å for adsorption). Therefore, its value should be better suited for procedures that prioritize pore accessibility rather than elevated surface reactivity.

**Figure 7:**
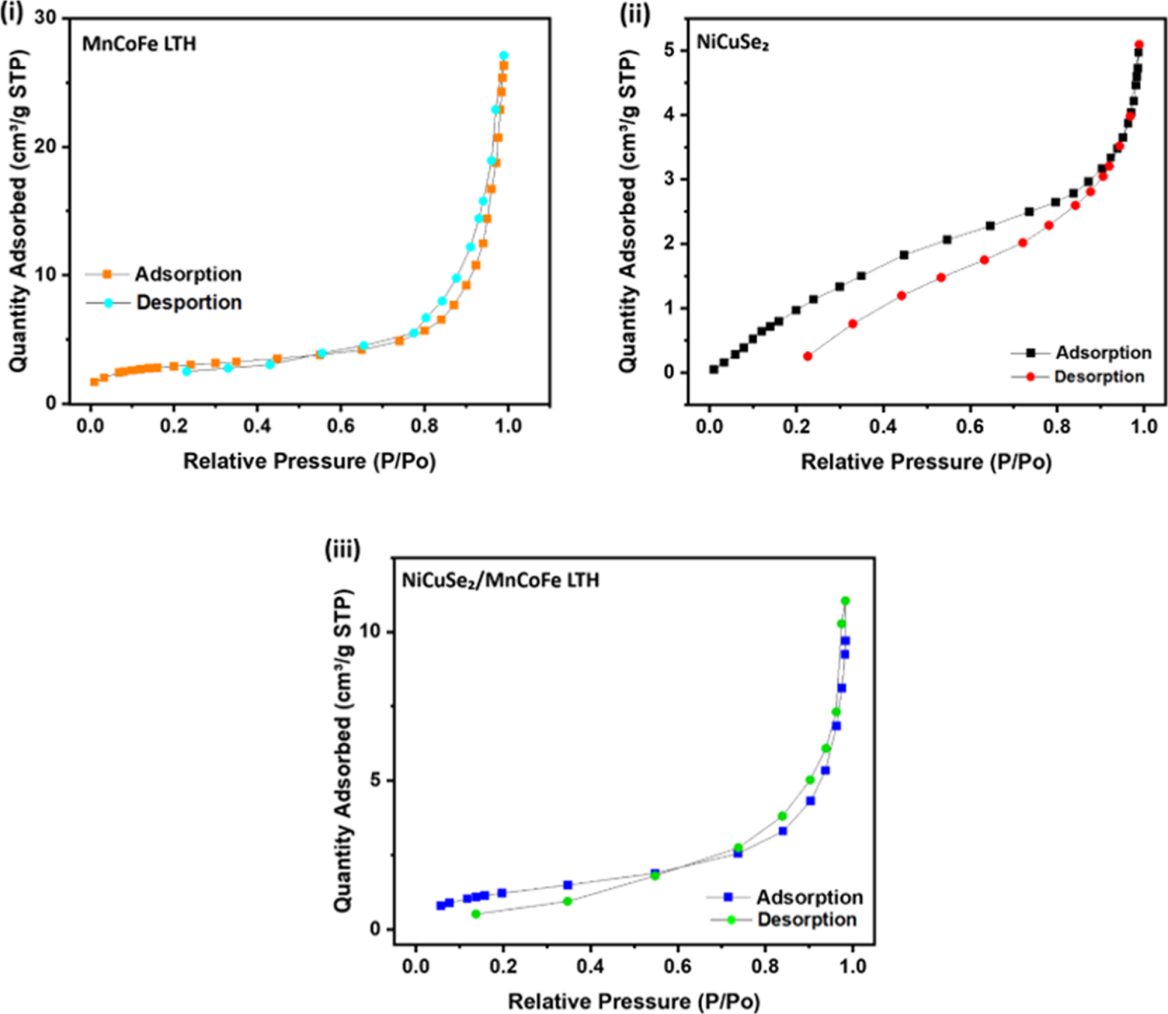
BET analyses of (i) MnCoFe LTH, (ii) NiCuSe_2_ and^26^ NiCuSe_2_/MnCoFe LTH nanocomposite.

The NiCuSe_2_/MnCoFe LTH nanocomposite showed a reduced surface area of 5.8 m^2^/g and increased average pore diameter of 129.63 Å for adsorption. This makes it excellent material for specialized applications requiring bigger pore sizes, perhaps improving capabilities in controlled release systems or analogous applications that need effective diffusion.

### 3.2 Electrochemical Characterization of NiCuSe_2_/MnCoFe LTH/GCE

The CV and EIS methods were employed to evaluate the surface properties of the unmodified/GCE and NiCuSe_2_/MnCoFe LTH/GCE electrodes. A solution of [Fe­(CN)_6_]^−3^/^–4^ (5.0 mM) in 0.1 M potassium chloride served as the electrolyte for this analysis. Figure 8A. illustrates the CV profiles of the unmodified and NiCuSe_2_/MnCoFe LTH-modified GCE electrodes (50 mV s^–1^). The peak-to-peak separation (ΔEp) was measured to be 0.21 V for the unmodified GCE,^27^ indicating a sluggish electron transfer process and limited electrochemical reversibility. In contrast, the NiCuSe_2_/MnCoFe-LTH/GCE exhibited a significantly smaller ΔEp of 0.1 V, reflecting enhanced charge transfer kinetics due to the synergistic effect of the nanocomposite structure. This substantial decrease in ΔEp demonstrates that the surface modification effectively facilitated faster electron exchange at the electrode–electrolyte interface, leading to improved electrochemical response compared to the unmodified electrode.

**Figure 8:**
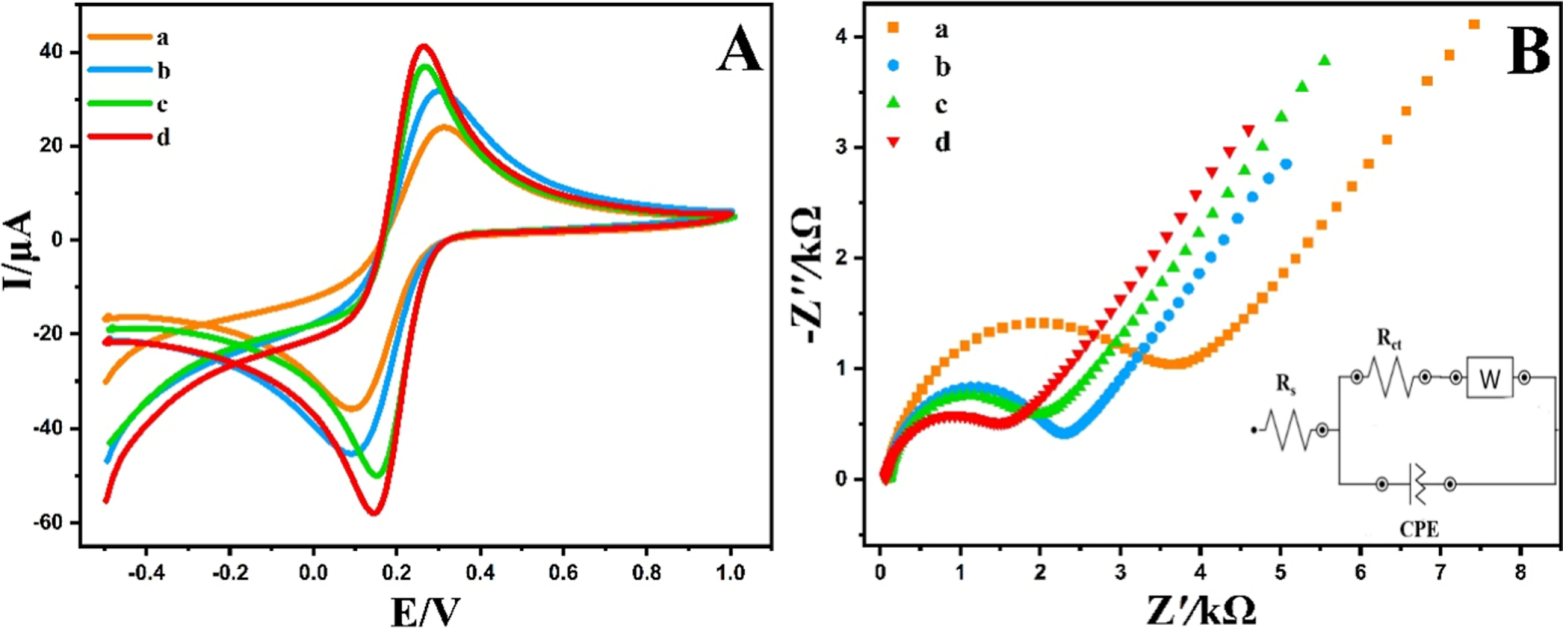
(A) CV curves at a scan rate of 50 mV s^–1^, and (B) EIS spectra of the bare GCE (a), NiCuSe_2_/GCE (b), MnCoFe LTH/GCE (c), and NiCuSe_2_/MnCoFe LTH/GCE (d) using 5 mM [Fe­(CN_6_)]^−3/–4^ as a redox probe versus Ag/AgCl reference electrode.

Furthermore, the NiCuSe_2_/MnCoFe LTH-modified GCE exhibited increased peak currents, indicating the strong electrocatalytic activity of the developed sensor.

Figures S1–S4 displays the CVs at various scan rates (25–250 mV). And the linear plot demonstrating the correlation between the peak current (*I*pa) and the square root of the scan rate (*v* ^1/2^) on the bare and different modified electrodes.

Using the Randles–Sevcik eq (eq 1), the slopes of the *I*pa vs *v* ^1/2^ plots and the diffusion coefficient (*D*) value for [Fe­(CN)_6_]^−3/–4^ (7.6 × 10^–6^ cm^2^ s^–1^), the electroactive surface areas were calculated.

1 $$I=\left(2.69\times 10^{5}\right)n^{3/2}AD^{1/2}v^{1/2}C_{0}$$

*A* represents the electrode area in cm^2^, *D* shows the diffusion coefficient (cm^2^/s), n is the number of electrodes (*n* = 1), *v* exhibits the potential scan rate (V/s), and *C*_0_ is the concentration (mol/cm^3^).

The EASA values were determined to be 0.075, 0.11, 0.15, and 0.16 cm^2^ for the unmodified GCE, NiCuSe_2_/GCE, MnCoFe LTH/GCE, and NiCuSe_2_/MnCoFe LTH/GCE, respectively. The 2.13-fold increase in EASA for the developed electrode highlights the exceptional properties of the NiCuSe_2_/MnCoFe LTH, providing a larger surface area and enhancing the current signal.

EIS was used to investigate the interfacial properties of different electrodes with a potential of 0.1 V and a frequency range of 10 kHz–0.1 Hz. The Nyquist plots for the unmodified GCE (a), NiCuSe_2_/GCE (b), MnCoFe LTH/GCE (c), and NiCuSe_2_/MnCoFe LTH/GCE (d) in a 5 mM solution of [Fe­(CN)_6_]^−3^/^–4^ in 0.1 M KCl are presented in Figure 8B. The EIS data were fitted to the corresponding Randles equivalent circuit, as illustrated in Figure 6B. In this circuit, R_s_ represents the electrolyte solution resistance, Rct corresponds to the charge transfer resistance, CPE denotes the constant phase element, and W accounts for the Warburg impedance caused by ion diffusion. The electrochemical parameters obtained from EIS after fitting with the Randles equivalent circuit are summarized in Table 1. The charge transfer resistance (*R*_ct_), which represents the resistance associated with charge transfer processes at the electrode interface, governs the electron transfer kinetics. The semicircle diameter in the Nyquist plot indicates the Rct value, with larger semicircles corresponding to slower electron transfer kinetics. *R*_ct_ is influenced by the dielectric and insulating properties of the electrode/electrolyte interface, which vary based on surface modifications. The Rct values were found to be 3.34, 2.09, 1.73, and 1.5 kΩ for the unmodified GCE, NiCuSe_2_/GCE, MnCoFe LTH/GCE, and NiCuSe_2_/MnCoFe LTH/GCE, respectively. The significantly lower *R*_ct_ of the NiCuSe_2_/MnCoFe LTH/GCE indicates enhanced conductivity and electron transfer efficiency, reflecting faster electron transfer kinetics. This improvement is attributed to the electrodeposition of NiCuSe_2_/MnCoFe LTH, which reduces interfacial resistance and increases electron exchange by providing a conductive matrix with abundant electroactive sites, enabling easier access of [Fe­(CN)_6_]^−3^/^–4^ to the electrode surface.

**Table 1:** EIS Derived Physical Parameters After Proper Fitting by Randle’s Equivalent Circuit

sensor	*R*_s_ (Ω)	*R*_ct_ (kΩ)	W (μMho*ŝ(1/2))	CPE (μMho*ŝN)
bare GCE	76.8	3.34	217	2.10 *N* = 0.85
NiCuSe2/GCE	415	2.09	304	0.874 *N* = 0.834
MnCoFe LTH/GCE	106	1.73	219	1.57 *N* = 0.864
NiCuSe2/MnCoFe LTH/GCE	67.2	1.5	286	4.22 *N* = 0.798

### 3.3 Comparison of the Electrochemical Behavior of POMA On the Bare/GCE and NiCuSe_2_/MnCoFe LTH/GCE

To assess the electrochemical behavior of POMA on various electrodes, DPV (modulation amplitude: 0.05 V, modulation time: 0.01 s, step potential: 0.005 V) and CV (scan rate: 50 mV/s) measurements were conducted in PBS (0.1 M, pH 4.0) in the presence of 40 μM POMA at both the bare GCE and NiCuSe_2_/MnCoFe LTH/GCE. The results are shown in Figure 9A (DPV) and Figure 9B (CV). Curve (a) indicates that no noticeable redox peaks were observed for NiCuSe_2_/MnCoFe LTH/GCE in the blank PBS solution. Upon the addition of POMA, a weak oxidation peak current (*I*pa) appeared at the bare GCE (curve b). In contrast, NiCuSe_2_/MnCoFe LTH/GCE displayed a pronounced oxidation peak with a lower oxidation potential, suggesting a significant electrocatalytic impact of the developed sensor on the electrochemical oxidation of POMA. Additionally, at the same concentration, the oxidation peak current of POMA obtained using DPV was higher than that recorded with CV. Therefore, the DPV method was selected for subsequent experiments.

**Figure 9:**
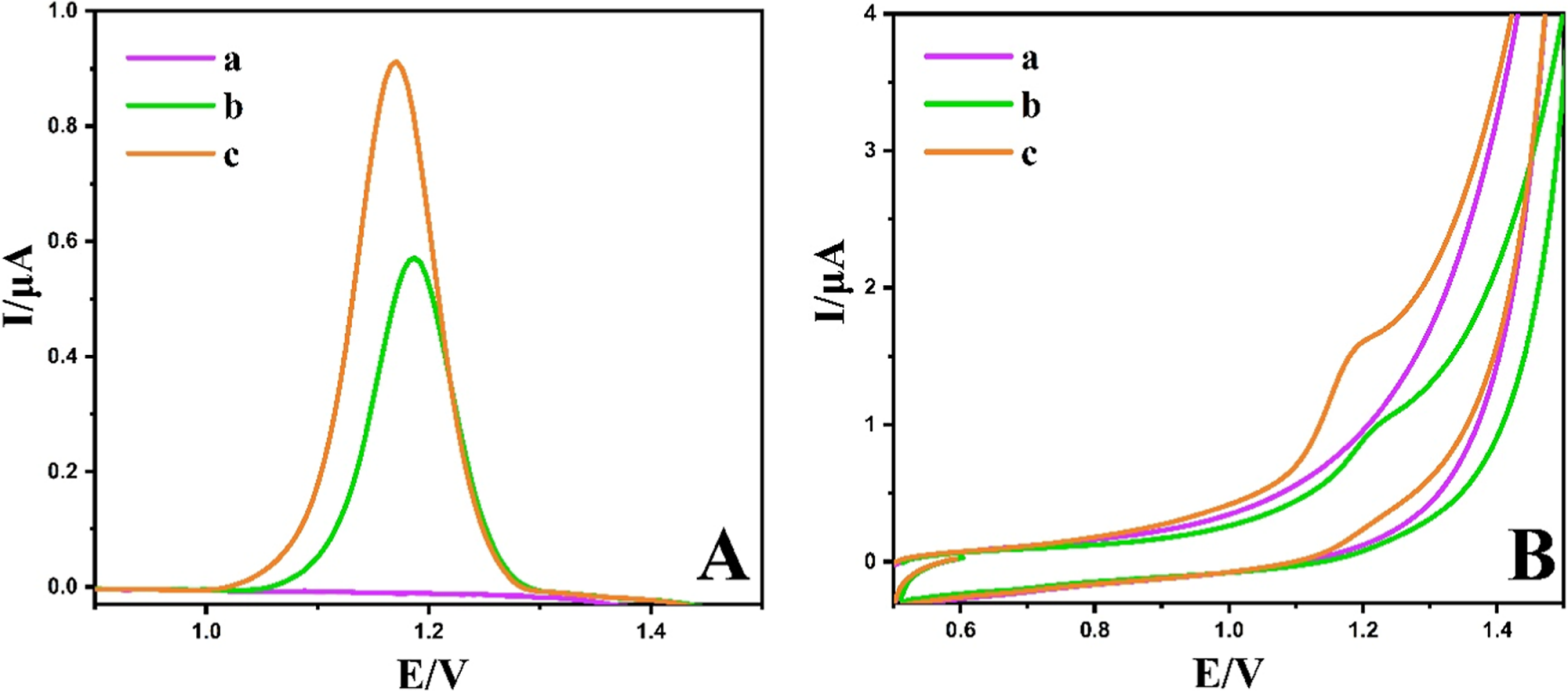
(A) DPV and (B) CV curves (50.0 mV/s) of 40 μM POMA on the blank (a), unmodified GCE (b), and NiCuSe_2_/MnCoFe LTH/GCE (c) vs Ag/AgCl reference electrode.

Furthermore, to compare the electrochemical response of POMA at different electrode surfaces, DPV measurements were performed for 40 μM POMA on the blank (curve a), unmodified GCE (curve b), MnCoFe LTH/GCE (curve c), NiCuSe_2_/GCE (curve d), and NiCuSe_2_/MnCoFe LTH/GCE (curv e) vs Ag/AgCl reference electrode (Figure S5). The most prominent oxidation peak of POMA was observed at the surface of NiCuSe_2_/MnCoFe LTH/GCE, which can be attributed to the synergistic effect of NiCuSe_2_ and MnCoFe LTH, enhancing the electrocatalytic activity of the modified electrode.

### 3.4 Effect of Supporting Electrolyte and pH

Buffers used as supporting electrolytes play a crucial role in the electro-oxidation of species on the surface of the modified GCE. Thus, selecting the most suitable buffer is necessary before optimizing other experimental parameters. To identify the optimal buffer for POMA detection, DPV measurements were performed with 40.0 μM POMA in different buffers, including Britton-Robinson (BR), phosphate (PBS), KCl, HCl, and NaOH (Figure S6A). The results indicated that the highest anodic peak current (*I*pa) was obtained in PBS. Based on these findings, 0.1 M PBS was selected as the optimal buffer for subsequent analyses.

The pH of the supporting electrolyte can influence the reaction mechanism and alter the peak potential and peak current of the target species. Thus, DPV analysis were conducted at different pH levels of PBS to gain insight into the oxidation mechanism of POMA at the NiCuSe_2_/MnCoFe LTH/GCE surface. As can be shown in Figure 10A, the maximum analytical response was achieved at pH 4, which was selected as the optimal pH for further experiments. A plot of pH versus oxidation potential (Figure 10B) revealed a linear relationship, with the potential shifting negatively as pH increased. Utilizing the Nernst equation of *E* = *E* ^θ^ – [(2.303 mRT)/(nF)] pH, the m/n (proton number/electron number) ratio was determined to be 0.5, signifying that the number of protons and electrons involved is half in this reaction.^28^

**Figure 10:**
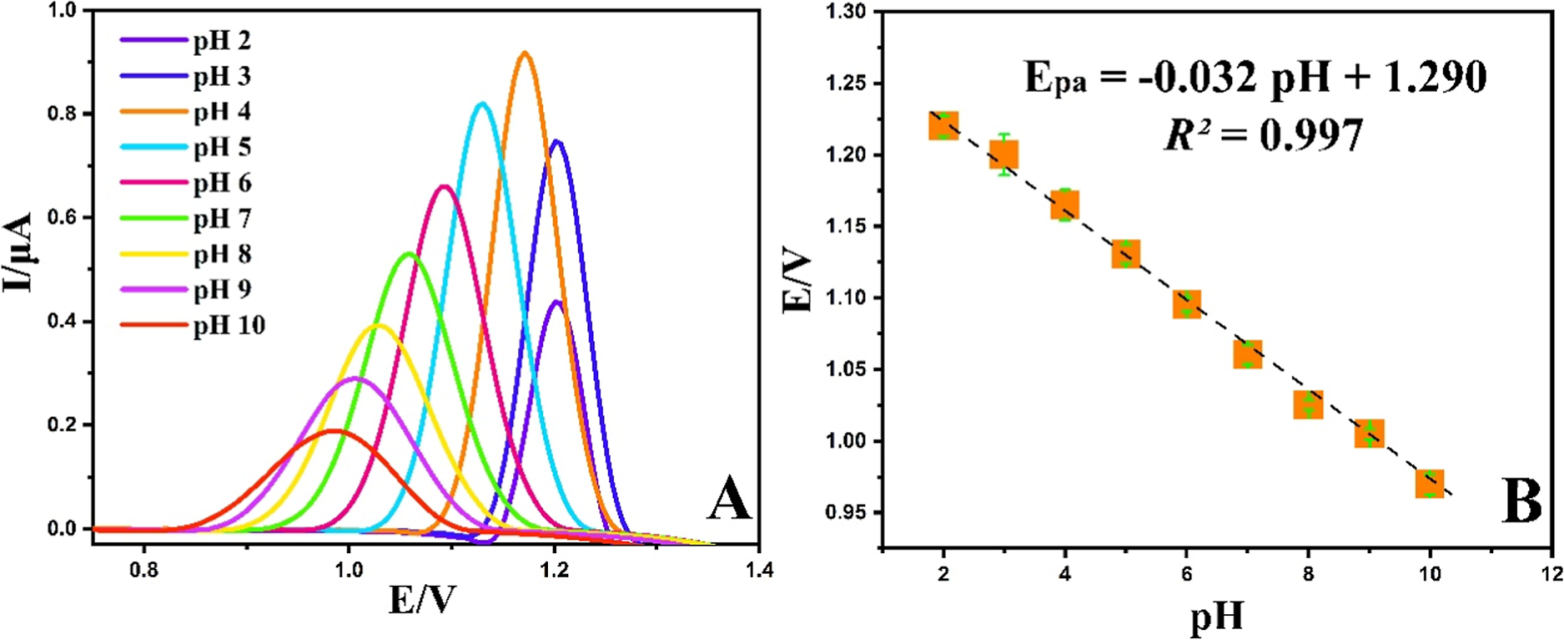
(A) DPV voltammograms of 40 μM POMA at various pH (2.0–10.0) of PBS (0.1 M) on the NiCuSe_2_/MnCoFe LTH/GCE and (B) the impact of pH on the peak potential (Epa) of POMA vs Ag/AgCl reference electrode.

### 3.5 Study of the NiCuSe_2_/MnCoFe LTH Modifier Amount and Concentration

The electrochemical response of POMA on the NiCuSe_2_/MnCoFe LTH/GCE was found to be influenced by the concentration of NiCuSe_2_/MnCoFe LTH on the electrode surface. To investigate this, varying concentrations of NiCuSe_2_/MnCoFe LTH (up to 0.5 mg/mL) were applied, and the electrochemical behavior of 40 μM POMA was assessed (Figure S5B). The results indicated that the electrochemical response of POMA enhanced as the concentration of NiCuSe_2_/MnCoFe LTH increased, reaching an optimum at 0.5 mg/mL. Beyond this concentration, no further improvement in the electrochemical response was observed, suggesting a saturation point. Consequently, 0.5 mg/mL was selected as the optimal modifier concentration.

The effect of varying amounts of NiCuSe_2_/MnCoFe LTH (ranging from 3.0 to 8.0 μL) on the voltammetric behavior of 40 μM POMA at pH 4.0 was investigated (Figure S5C). The results demonstrated that as the volume of NiCuSe_2_/MnCoFe LTH increased, the electrochemical response initially improved, but beyond an application of 3 μL, the current began to decrease. This suggests that excessive amounts of the composite may lead to undesirable effects on the electrode surface, possibly due to overcoverage or altered electroactive site accessibility. Based on these findings, 3 μL was selected as the optimal amount of NiCuSe_2_/MnCoFe LTH for subsequent experiments to ensure the best electrochemical performance.

### 3.6 Effect of Scan Rate

The impact of scan rate on the peak current (*I*pa) and peak potential (Epa) at the NiCuSe_2_/MnCoFe LTH/GCE was studied using cyclic voltammetry in PBS (0.1 M, pH = 4.0) containing 40 μM POMA at varying scan rates ranging from 25 to 250 mV s^–1^ (Figure 11A). A positive shift in the Epa with increasing scan rate was observed, indicating an irreversible electrochemical reaction.^29^ As can be seen in Figure 11B, the anodic peak current (*I*pa) exhibited a linear relationship with the square root of the scan rate (*I*pa = 0.037 + 0.001, *R* ^2^ = 0.998), confirming that the oxidation process on the NiCuSe_2_/MnCoFe LTH-modified GCE surface is diffusion-controlled. Additionally, the plot of log (*I*pa) versus log (υ) was found to be linear (Figure 11C), with a slope value close to the theoretical value of 0.5, further corroborating a diffusion-controlled electrode process.^30^

**Figure 11:**
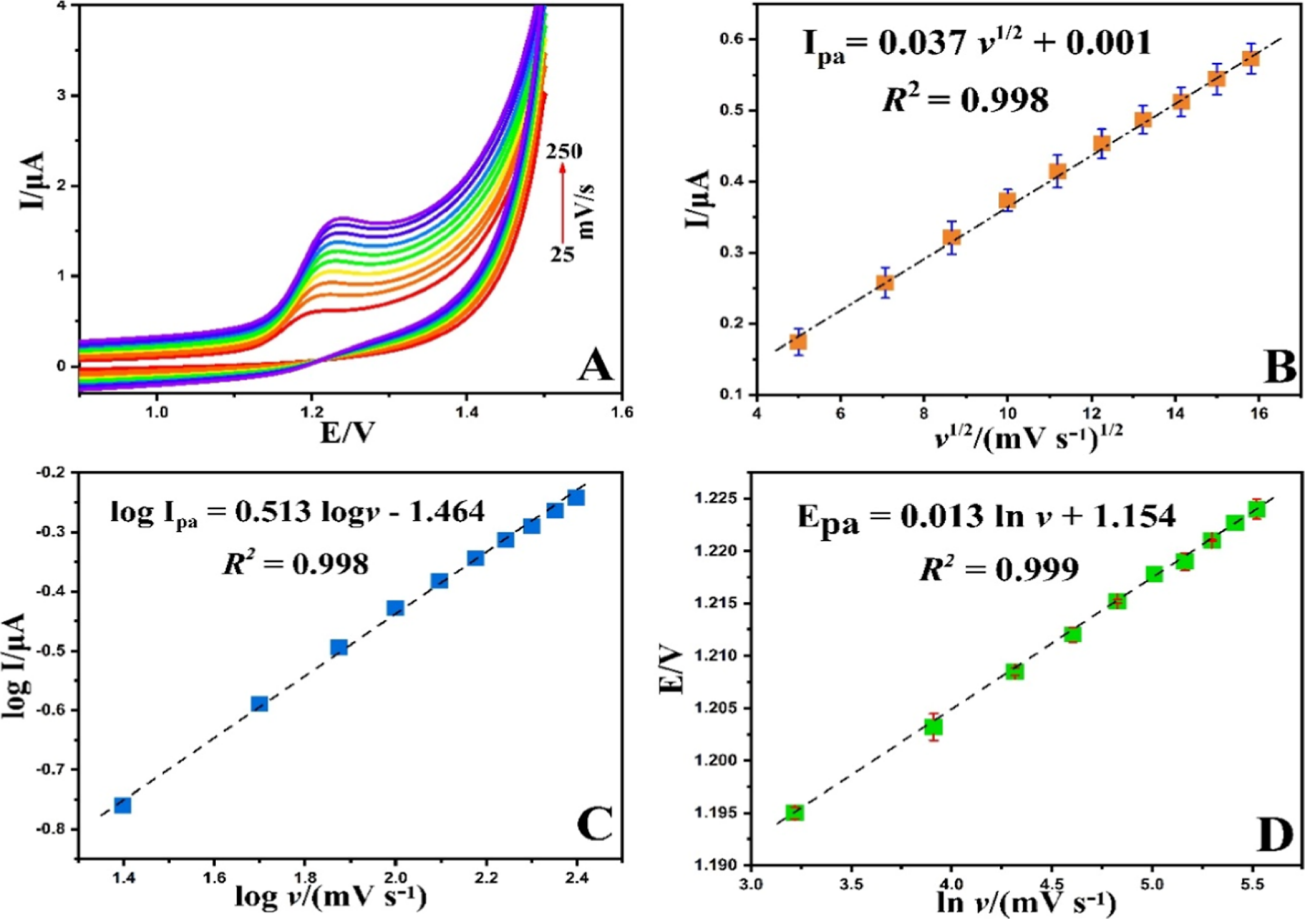
(A) Scan rate effect of 40 μM POMA in PBS (0.1 M, pH = 4.0), (B) plot of *I*pa (μA) vs *v* ^1/2^ (mVs^–1^)^1/2^, (C) plot of log *I*pa (μA) vs log *v* (mVs^–1^) and (D) plot of Epa (V) vs ln *v* (mVs^–1^) vs Ag/AgCl reference electrode.

Furthermore, the peak potential (Epa) showed a linear relationship with the ln of the scan rate (ln υ), expressed as Epa = 0.013 ln υ + 1.154 (*R* ^2^ = 0.999) (Figure 11D). The number of electrons (*n*) involved in the process was calculated to be 2 based on the slope of the Epa versus ln υ plot, in accordance with Laviron’s theory.^31^ As previously indicated, the total number of electrons and protons participating in the charge transfer was determined to be half, signifying that two electrons and one proton are involved in the oxidation of POMA at the NiCuSe_2_/MnCoFe LTH/GCE surface. The plausible reaction pathway for the electrooxidation of POMA on NiCuSe_2_/MnCoFe LTH/GCE in 0.1 M PBS solution (pH 4.0) is proposed in Scheme 1. In the oxidation process of POMA, the NH_2_ group on the six-membered heterocyclic ring (aniline) undergoes oxidation. As described in recent studies, one hydrogen atom is released from the NH_2_ group, resulting in a resonance effect on the ring. This explains the observed proton to electron ratio of 1:2 during the oxidation process, consistent with the oxidation mechanism outlined in the literature.^32^

**Scheme 1:**
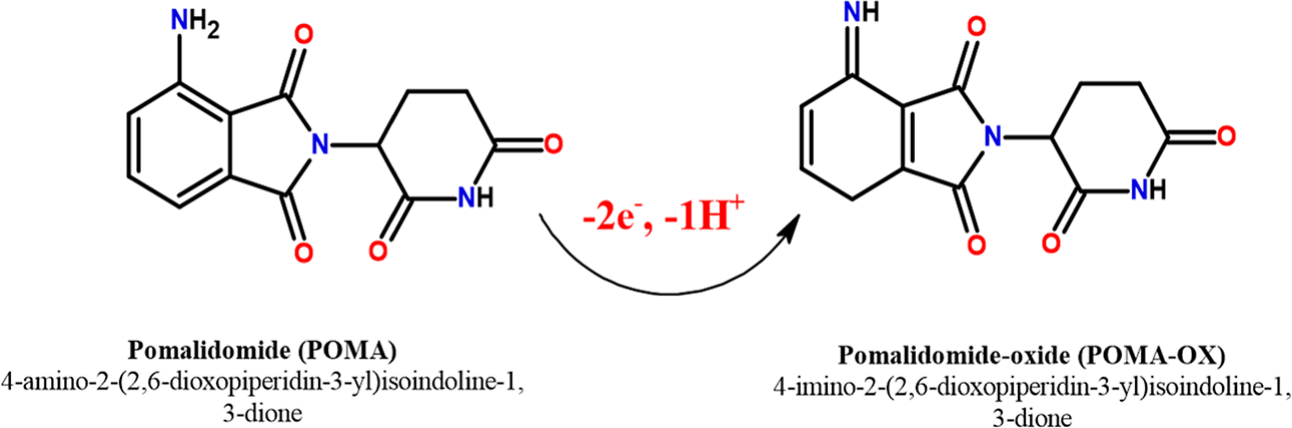
Plausible Electrooxidation Process of POMA

The surface coverage concentration of the developed NiCuSe_2_/MnCoFe LTH/GCE was calculated using the Laviron’s (eq 2).^33^ The value of the surface coverage concentration (Γ) on the NiCuSe_2_/MnCoFe LTH-modified electrode was found to be 3.33 × 10^–9^ M cm^–2^.

2 $$Ipa=\frac{n^{2}F^{2}A\Gamma }{4RT}v$$

As depicted in Figure S7, the chronoamperometric data present a comprehensive collection of experimental findings involving different concentrations of POMA (100.0, 200.0 μM) in the PBS buffer (0.1 M, pH 4). The main aim of these tests was to determine the diffusion coefficient (D) of POMA at the NiCuSe_2_/MnCoFe LTH/GCE surface, which was accomplished using the Cottrell equation.^34^ Linear curves were generated by graphing the current (*I*) against the reciprocal of the square root of time (*t* ^–1/2^), originating from the various concentrations of POMA. The diffusion coefficient was calculated as 3.8 × 10^–5^ cm^2^/s, demonstrating superior electrocatalytic oxidation.

### 3.7 Analytical Performance

#### 3.7.1 Quantification of POMA at the Surface of NiCuSe_2_/MnCoFe LTH/GCE

In this study, the NiCuSe_2_/MnCoFe LTH/GCE was utilized to determine various concentrations of POMA using the DPV method under optimal conditions (PBS at pH 4.0). Within the linear concentration range of 0.02–10.3 μM, the oxidation current of POMA exhibited a proportional increase with rising drug concentrations (Figure 12). The linear regression equation was found to be *I*pa (μA) = 0.058 C_POMA_ (μM) + 0.041, *R* ^2^ = 0.997. Additionally, the limit of detection (LOD) was found as 4.7 nM utilizing the formula LOD = 3σ/*S* where σ represents the standard deviation of ten measurements for the blank solution and *S* is the slope of the calibration curve.^35^ These results demonstrate the high sensitivity of the NiCuSe_2_/MnCoFe LTH/GCE for POMA detection.

**Figure 12:**
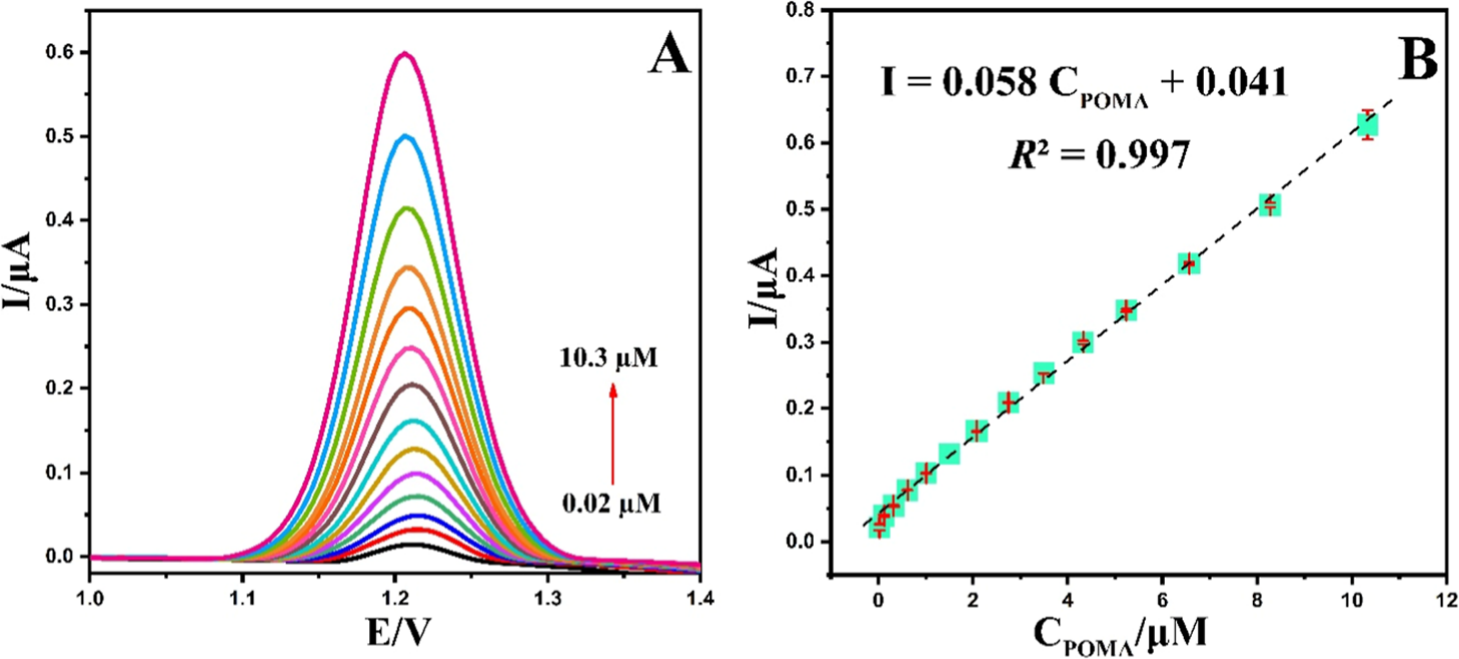
(A) DPVs of NiCuSe_2_/MnCoFe LTH/GCE in PBS (0.1 M, pH = 4.0) containing various concentrations of POMA (0.02–10.3 μM); (B) the relationship between *I*pa and POMA concentration vs Ag/AgCl.

Table 2 presents a comparative analysis of POMA quantification, assessing the proposed sensor alongside the reported method based on key analytical parameters, including the detection method, electrode modification, linear range, LOD, and applicability to real samples.

**Table 2:** Comparison Between the Proposed Method for Determination of POMA to Previously Reported Methods

method	modifier	linear range (μM)	LOD (μM)	Application	ref
SWV	MIP/Fe_3_O_4_/MoS_2_/GCE	0.001–0.2	0.00033	human plasma and capsules.	^32^
DPV	NiCuSe_2_/MnCoFe LTH/GCE	0.02–10.3	0.0047	human serum, human urine and capsules.	our work

#### 3.7.2 The Repeatability, Reproducibility, and Interference Study of the NiCuSe_2_/MnCoFe LTH/GCE

The performance of the NiCuSe_2_/MnCoFe LTH/GCE was validated by assessing its repeatability and reproducibility through DPV measurements in a 0.1 M phosphate buffer solution (pH 4.0) containing 2 μM POMA. Repeatability was evaluated by conducting 12 consecutive measurements of 2 μM POMA utilizing the same electrode (Figure S8), while reproducibility was assessed by comparing DPV responses obtained from nine independently prepared sensors (Figure S9). The sensor demonstrated excellent stability, with relative standard deviations (RSD) of 1.11% for repeatability and 1.02% for reproducibility, confirming its reliable performance.

One of the most substantial necessities for applying a high-performance sensor is the ability of the modified electrode to distinguish between the analyte and the other species present in the analysis matrix. To evaluate the selectivity of the developed sensor, it was inserted into the solutions of POMA in the presence of various coexisting interference substances commonly found in biological and pharmaceutical samples. Initially, 2 μM POMA was introduced into a 0.1 M PBS (pH 4.0), and the DPV curve was recorded, as shown in Figure S10. Subsequently, sodium sulfate (Na_2_SO_4_), potassium chloride, d-glucose, l-arginine, l-methionine, potassium nitrate, uric acid, ascorbic acid, dopamine, and paracetamol, were added at 100-fold higher concentrations. The results in Figure S10 demonstrate that these interfering agents caused less than a 2% change in the peak current of POMA. These findings indicate that the NiCuSe_2_/MnCoFe LTH/GCE exhibits satisfactory selectivity for POMA determination.

#### 3.7.3 Determination of POMA in Biological and Pharmaceutical Samples

The performance of the developed sensor was assessed by measuring the concentrations of POMA in human blood serum, human urine, and POMA capsules using the DPV method, and calculating the recovery values. The results are summarized in Table 3. It is clear from this table that the recovery study of POMA through the voltammetric method lies in the range of 98.48% to 101.5%. The RSD for the method proposed here was less than 3%. All these studies confirm that this sensor is reliable and accurate for the analytical quantification of POMA in real samples.

**Table 3:** Measurement of POMA in Human Blood Serum, Human Urine, and Capsules

sample	added (μM)	found (μM)^a^	RSD (%)	Recovery (%)
human blood serum	2 4 6	2.02 ± 0.001^b^ 3.97 ± 0.004 5.91 ± 0.005	1.0 1.7 1.5	101.1 99.19 98.53
human urine	2 4 6	2.02 ± 0.004 3.98 ± 0.004 5.99 ± 0.003	2.6 1.6 1.1	101.2 99.23 99.89
capsule	2 4 6	2.06 ± 0.003 3.93 ± 0.002 6.02 ± 0.009	2.3 1.2 2.7	101.5 98.48 100.3

a Average of three replicate measurements.

b Mean ± standard deviation for *n* = 3.

## 4 Conclusion

In this study, a novel NiCuSe_2_/MnCoFe LTH nanocomposite-modified GCE was developed for the electrochemical detection of POMA. The structural and morphological characterization confirmed the successful synthesis of the composite, while electrochemical studies using CV and EIS highlighted its excellent conductivity, impressive electrocatalytic activity, and rapid electron transfer kinetics. The developed sensor exhibited remarkable analytical performance, including a wide linear detection range of 0.02–10.3 μM and an ultralow detection limit of 4.7 nM, as determined via DPV. The sensor demonstrated excellent repeatability (RSD = 1.11%) and reproducibility (RSD = 1.02%), as well as robust selectivity, with less than a 2% peak current deviation in the presence of common interfering substances. Furthermore, its application to real samples, including POMA capsules, human blood serum, and urine, yielded recovery rates between 98.48% and 101.5%, with RSD values below 3%, confirming the sensor’s reliability and accuracy in practical scenarios. These results establish the NiCuSe_2_/MnCoFe LTH/GCE as a powerful and versatile tool for the sensitive and selective quantification of POMA in pharmaceutical and biological samples, offering promising potential for broader applications in electrochemical drug detection. Despite promising results, potential limitations of the sensor include its long-term stability, performance under varying storage conditions, and challenges in complex real sample matrices. Future work will focus on improving sensor robustness in diverse samples, assessing large-scale production feasibility, and integrating the sensor into portable devices for on-site applications. These efforts will enhance the sensor’s practical applicability in electrochemical drug detection.

## Supplementary Material



## Notes

Additional experimental details include materials, reagents, apparatus, details on the effect of concentration and amount of composite, repeatability, reproducibility, selectivity, cyclic voltammogram, and differential pulse voltammograms (PDF)
