# Supplementary material for: Highly Sensitive Electrochemical Detection of Pomalidomide Using a Novel NiCuSe2/MnCoFe LTH Nanocomposite Sensor: Application to Real Sample Analysis
Source: ACS Omega. 2025 Jun 20;10(25):26605–17. doi: 10.1021/acsomega.5c00694 (PMC12223861; doi:10.1021/acsomega.5c00694)
Supplement: Supplementary file 1 [file ao5c00694_si_001.pdf]

Supporting Information

Highly Sensitive Electrochemical Detection of Pomalidomide Using a Novel $NiCuSe_2$@MnCoFe LTH Nanocomposite Sensor: Application to Real Sample Analysis

Wiem Bouali [a b*], ***Asena Ayse Genc*** [a b], ***Nevin Erk*** [a*], ***Hassan Elzain Hassan AHMED*** [c], *and* ***Mustafa Soylak*** [c d e]

[a] Ankara University, Faculty of Pharmacy, Department of Analytical Chemistry, 06560 Ankara, Turkey

[b] Ankara University, The Graduate School of the Health Sciences, 06110 Ankara, Turkey

[c] Erciyes University, Faculty of Sciences, Department of Chemistry, 38039, Kayseri, Turkey

[d] Technology Research & Application Center (TAUM), Erciyes University, 38039, Kayseri, Turkey

[e] Turkish Academy of Sciences (TUBA), Cankaya, Ankara, Turkey

Email corresponding author: erk@pharmacy.ankara.edu.tr

wbouali@ankara.edu.tr

Materials and Reagents

In this study, Glucose (99.5 %), L-arginine (98.0 %), L-methionine, sodium hydroxide, potassium hexacyanoferrate (III) ($K_3Fe(CN)_6$, 99.5 %), hydrochloric acid, sodium Acetate, ascorbic acid, uric acid (99.0 %), acetic acid, potassium chloride, sodium phosphate, sodium sulfate, potassium chloride, sodium sulfate, were purchased from Sigma Aldrich Co. (https://www.sigmaaldrich.com, Germany). The stock solution of Pomalidomide was prepared in methanol: water (1:1). All chemical compounds were analytical grade and used without additional refinement.

Apparatus

Voltammetric experiments were carried out using AUTO LAB system with PGSTAT204 electrochemical workstation (Metrohm Inc., Switzerland) with a glassy carbon electrode system in a one-compartment of 10 mL electrochemical cell. All electrochemical measurements were performed at 25 °C unless otherwise specified.

The pH of the supporting electrolyte was monitored with a pH meter (Hanna Instruments, Woonsocket, Rhode Island, USA).

FT-IR experiments were conducted on a Perkin-Elmer FT-IR spectrometer model 400 from the United States. BRUKER AXS D8 ADVANCE from Germany was employed for the X-ray diffraction tests. We applied the ZEISS Crossbeam 550 from Germany for FE-SEM and FE-SEM-EDX characterizations. We used the Brunauer–Emmett–Teller (BET) gas auto-sorption (Micromeritics Gemini VII 2390) to study the porosity and surface area of the MnCoFe-LTH, NiCuSe, and NiCuSe/MnCoFe-LTH.

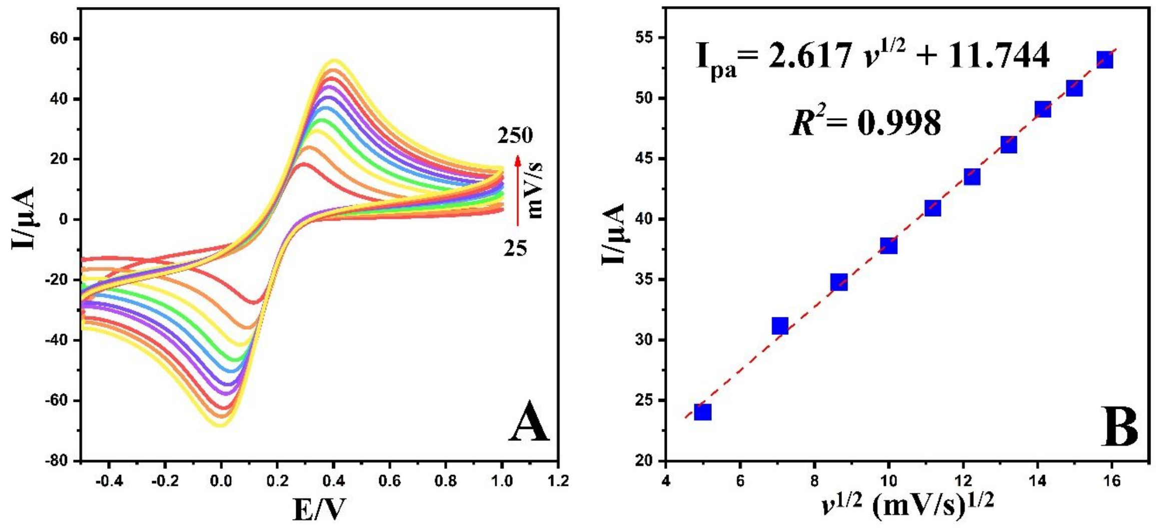

Figure S1. The recorded CV curves (A), and the relationship between I_{pa} vs. $v^{1/2}$ (B) on the bare GCE at various scan rates in 5.0 mM $[Fe(CN)_6]^{3-/4-}$ and 0.1 M KCl.

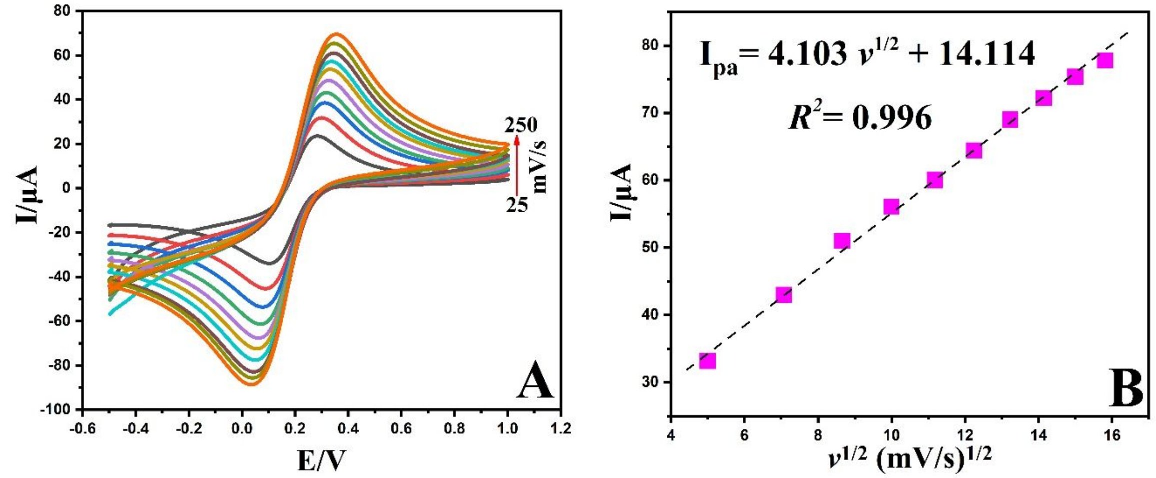

Figure S2. The recorded CV curves (A), and the relationship between I_{pa} vs. $v^{1/2}$ (B) on the $NiCuSe_2$/GCE at various scan rates in 5.0 mM $[Fe(CN)_6]^{3-/4-}$ and 0.1 M KCl.

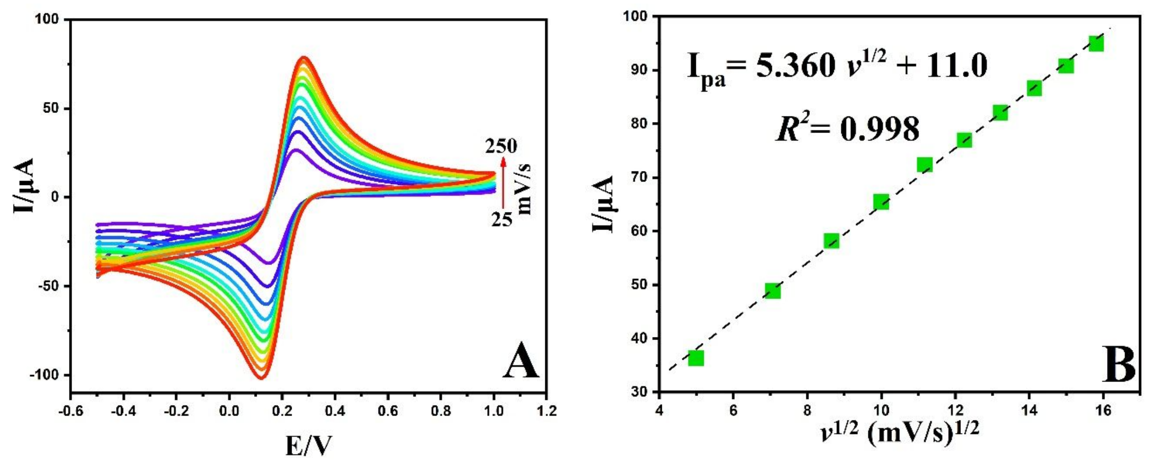

Figure S3. The recorded CV curves (A), and the relationship between I_{pa} vs. $v^{1/2}$ (B) on the MnCoFe LTH/GCE at various scan rates in 5.0 mM $[Fe (CN)_6]^{3-/4-}$ and 0.1 M KCl.

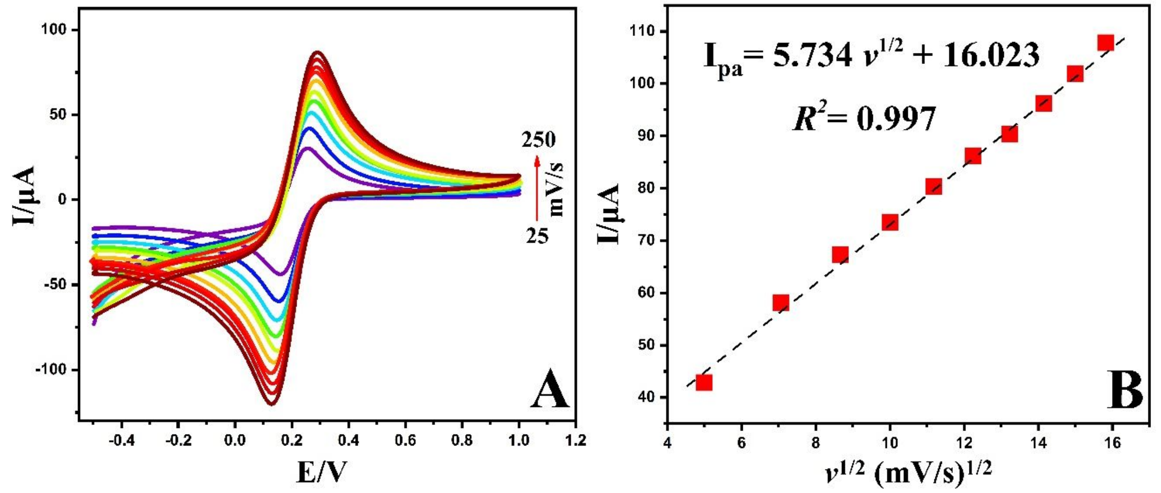

Figure S4. The recorded CV curves (A), and the relationship between I_{pa} vs. $v^{1/2}$ (B) on the $NiCuSe_2$@MnCoFe LTH/GCE at various scan rates in 5.0 mM $[Fe (CN)_6]^{3-/4-}$ and 0.1 M KCl.

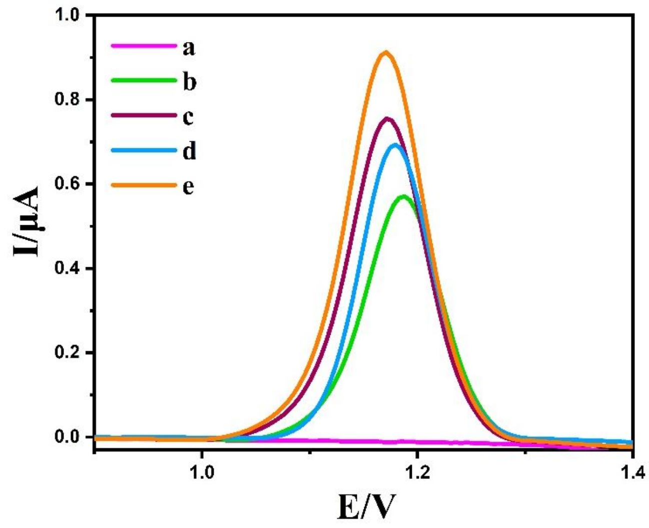

Figure S5. DPV of 40 μM POMA on the blank (a), unmodified GCE (b), MnCoFe LTH/GCE (c), $NiCuSe_2$/GCE (d), and $NiCuSe_2$/MnCoFe LTH/GCE (e) vs. Ag/AgCl reference electrode.

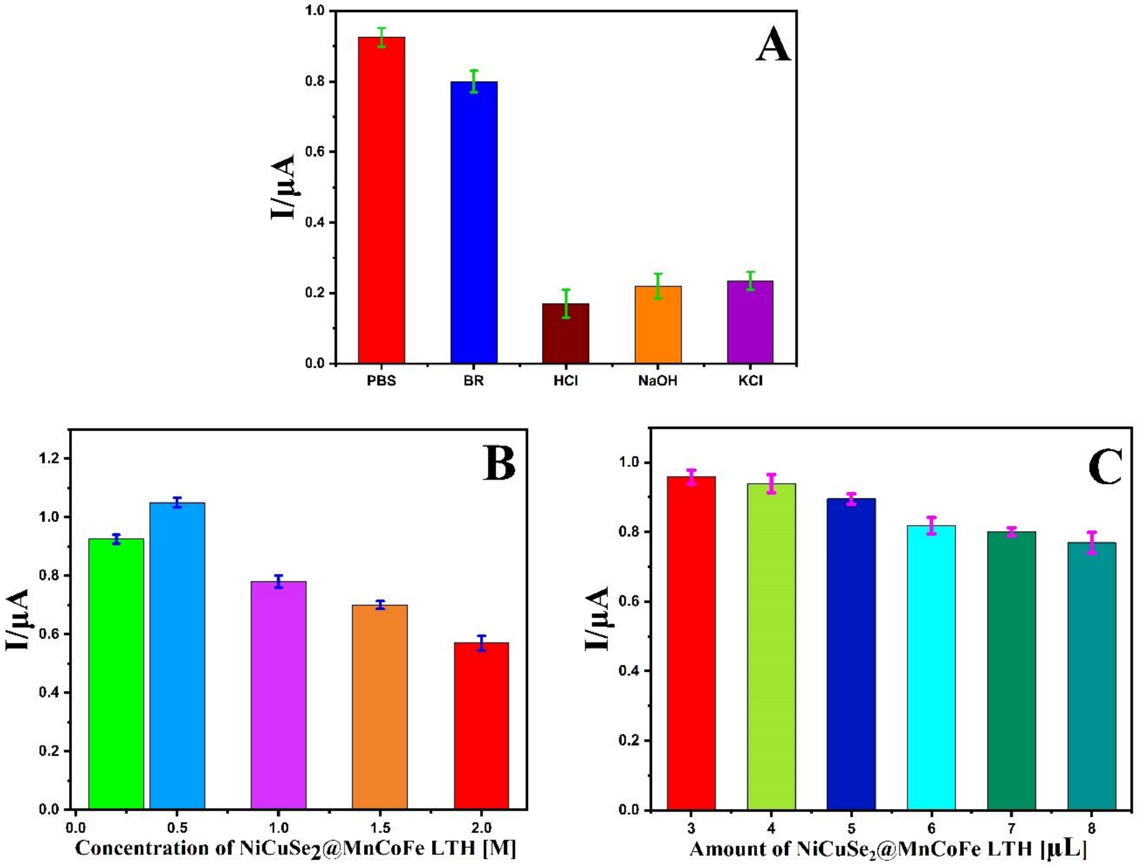

Figure S6. Influence of supporting electrolyte (A), concentration (B), and amount (C) of $NiCuSe_2$/MnCoFe LTH on the oxidation peak currents of 40 μM POMA.

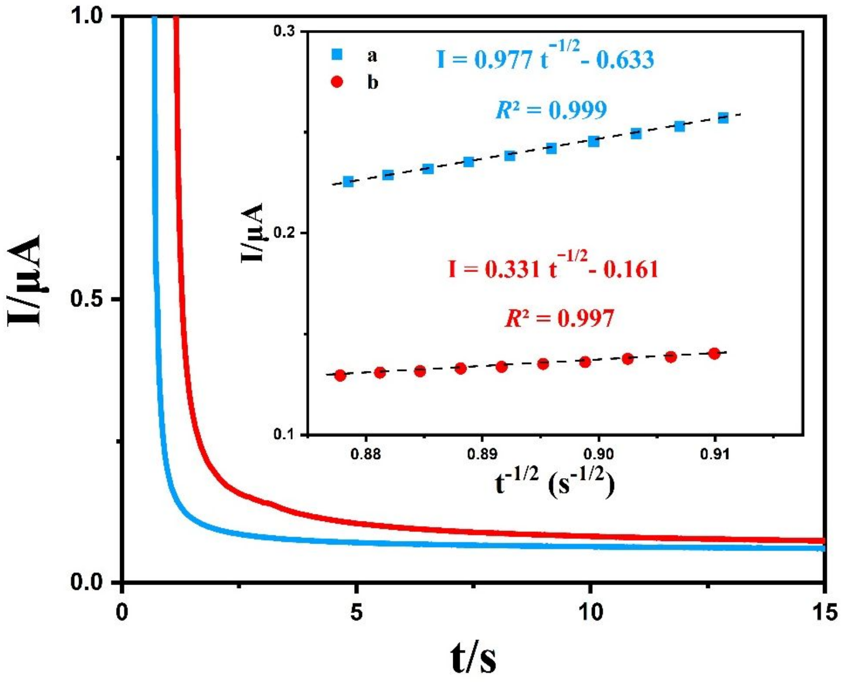

Figure S7. (A) Chronoamperograms obtained at $NiCuSe_2$/MnCoFe LTH/GCE in the presence of 100 (a) and 200 μM (b) POMA; (B) Cottrell's plot for the data from the chronoamperograms.

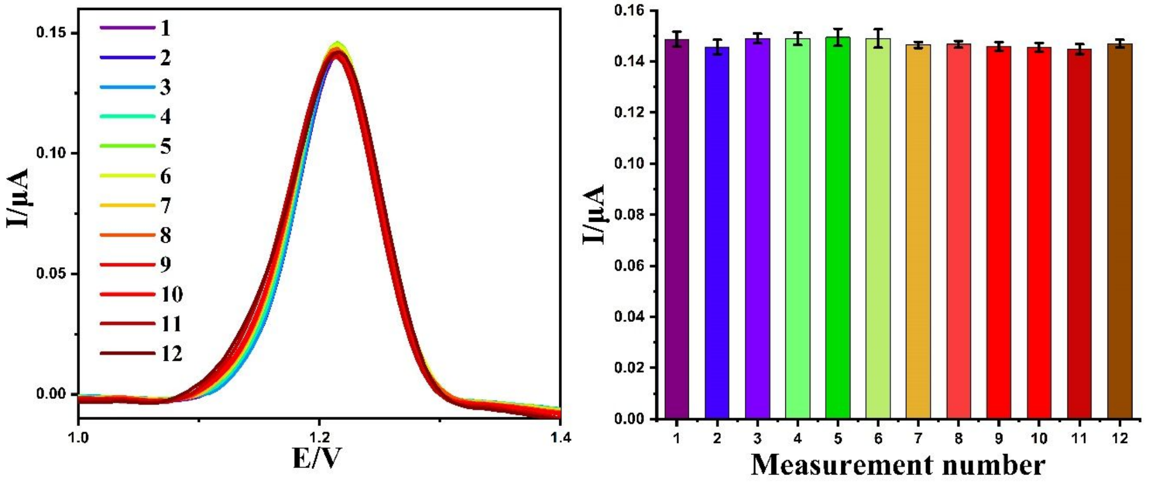

Figure S8. DPV curves and histogram of repeatability of $NiCuSe_2$/MnCoFe LTH/GCE in 2.0 μM POMA (PBS buffer, pH 4.0).

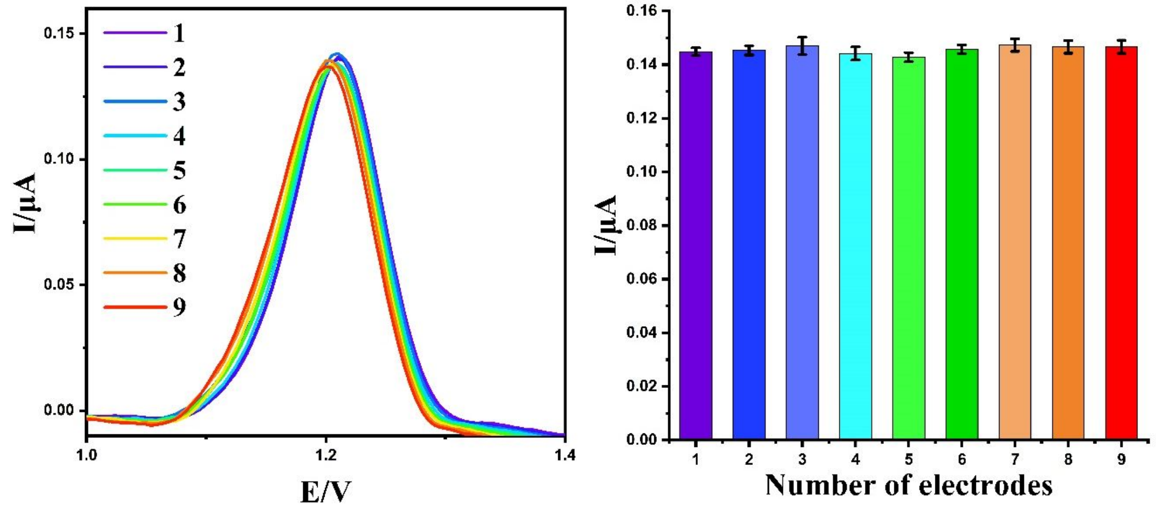

Figure S9. DPV curves and histogram of reproducibility of $NiCuSe_2$/MnCoFe LTH/GCE in 2.0 μM POMA (PBS buffer, pH 4.0).

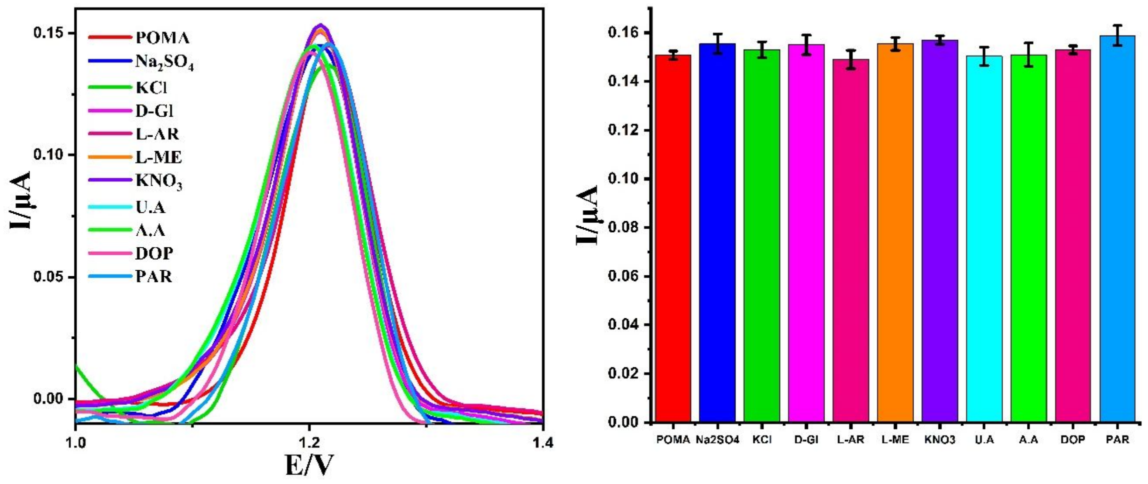

Figure S10. DPV curves and histogram of selectivity of $NiCuSe_2$/MnCoFe LTH/GCE in 2.0 μM POMA (PBS buffer, pH 4.0).
